# Association of State Medicaid Expansion With Racial/Ethnic Disparities in Liver Transplant Wait-listing in the United States

**DOI:** 10.1001/jamanetworkopen.2020.19869

**Published:** 2020-10-08

**Authors:** Lauren D. Nephew, Kelly Mosesso, Archita Desai, Marwan Ghabril, Eric S. Orman, Kavish R. Patidar, Chandrashekhar Kubal, Mazen Noureddin, Naga Chalasani

**Affiliations:** 1 Division of Gastroenterology and Hepatology, Department of Medicine, Indiana University School of Medicine, Indianapolis; 2 Department of Biostatistics, Indiana University Fairbanks School of Public Health and School of Medicine, Indianapolis; 3 Division of Organ Transplantation, Department of Surgery, Indiana University School of Medicine, Indianapolis; 4 Division of Gastroenterology and Hepatology, Department of Medicine, Cedars-Sinai Medical Center, Los Angeles, California

## Abstract

**Importance:**

Millions of Americans gained insurance through the state expansion of Medicaid, but several states with large populations of racial/ethnic minorities did not expand their programs.

**Objective:**

To investigate the implications of Medicaid expansion for liver transplant (LT) wait-listing trends for racial/ethnic minorities.

**Design, Setting, and Participants:**

A cohort study was performed of adults wait-listed for LT using the United Network of Organ Sharing database between January 1, 2010, and December 31, 2017. Poisson regression and a controlled, interrupted time series analysis were used to model trends in wait-listing rates by race/ethnicity. The setting was LT centers in the United States.

**Main Outcomes and Measures:**

(1) Wait-listing rates by race/ethnicity in states that expanded Medicaid (expansion states) compared with those that did not (nonexpansion states) and (2) actual vs predicted rates of LT wait-listing by race/ethnicity after Medicaid expansion.

**Results:**

There were 75 748 patients (median age, 57.0 [interquartile range, 50.0-62.0] years; 48 566 [64.1%] male) wait-listed for LT during the study period. The cohort was 8.9% Black and 16.4% Hispanic. Black patients and Hispanic patients were statistically significantly more likely to be wait-listed in expansion states than in nonexpansion states (incidence rate ratio [IRR], 1.54 [95% CI, 1.44-1.64] for Black patients and 1.21 [95% CI, 1.15-1.28] for Hispanic patients). After Medicaid expansion, there was a decrease in the wait-listing rate of Black patients in expansion states (annual percentage change [APC], −4.4%; 95% CI, −8.2% to −0.6%) but not in nonexpansion states (APC, 0.5%; 95% CI, −4.0% to 5.2%). This decrease was not seen when Black patients with hepatitis C virus (HCV) were excluded from the analysis (APC, 3.1%; 95% CI, −2.4% to 8.9%), suggesting that they may be responsible for this expansion state trend. Hispanic Medicaid patients without HCV were statistically significantly more likely to be wait-listed in the post–Medicaid expansion era than would have been predicted without Medicaid expansion (APC, 13.2%; 95% CI, 4.0%-23.2%).

**Conclusions and Relevance:**

This cohort study found that LT wait-listing rates have decreased for Black patients with HCV in states that expanded Medicaid. Conversely, wait-listing rates have increased for Hispanic patients without HCV. Black patients and Hispanic patients may have benefited differently from Medicaid expansion.

## Key Points

**Question:**

Was state Medicaid expansion associated with increased liver transplant wait-listing rates for racial/ethnic minorities?

**Findings:**

In this cohort study of 75 748 patients, wait-listing rates were higher in Black patients and Hispanic patients living in expansion states compared with those living in nonexpansion states, and there was a statistically significant decrease in the wait-listing rate of Black patients in expansion states after Medicaid expansion that was not seen when Black patients with hepatitis C virus were excluded from the analysis. Hispanic patients without hepatitis C virus were wait-listed at higher rates than would have been predicted without Medicaid expansion.

**Meaning:**

Black patients and Hispanic patients may have benefited differently from Medicaid expansion.

## Teaser

This cohort study investigates the implications of state Medicaid expansion for liver transplant wait-listing trends for racial/ethnic minorities.

## Introduction

Liver transplant (LT) is the standard-of-care and life-saving treatment for end-stage liver disease (ESLD). Although comprehensive national data do not exist on the exact burden of ESLD, data suggest racial/ethnic disparities in access to LT wait-listing.^1,2^ This finding is not entirely unexpected because disparities earlier in the care continuum have been described in access to hepatitis C virus (HCV) therapy, treatment for cirrhosis complications, and referral for LT.^3,4,5^ Once racial/ethnic minority patients are wait-listed for LT, Model for End-Stage Liver Disease era data suggest that transplant rates are similar to those of White candidates.^6^ Therefore, a critical step in achieving equitable care of patients with ESLD is ensuring access to LT wait-listing.

Financial screening is the first step in the LT evaluation process.^7^ This step poses a substantial barrier to many patients. More than one-quarter of transplant ethics consultations describe restrictions in transplant-related treatment for financial or insurance reasons.^8^

In 2013, approximately 44 million Americans were uninsured (40.5% of Hispanic individuals, 25.8% of Black individuals, and 14.8% of White individuals).^9,10^ In 2014, the Affordable Care Act (ACA) sought to expand insurance coverage through an optional state-level expansion of Medicaid.^11,12^ After the main ACA provisions went into effect in 2014, the percentage of adults who were uninsured decreased by 7.1% for Hispanic patients, 5.1% for Black patients, and 3% for White patients.^9^ Coverage gains were greater in states that expanded Medicaid programs.^9^ Several states that did not expand their Medicaid programs have large populations of racial/ethnic minorities.^13^

Analysis to date on the association of Medicaid expansion with LT wait-listing has included only 1 year of data before and after expansion, provided limited or no analysis of race/ethnicity, focused broadly on all solid organ transplantation, or used difference-in-differences methods that did not account for trends that were already under way before Medicaid expansion.^14,15,16^ We hypothesized that insurance status would be a barrier to LT wait-listing for racial/ethnic minorities and that Medicaid expansion would be associated with higher wait-listing rates for racial/ethnic minorities living in states that expanded Medicaid.

## Methods

### Study Population and Design

A cohort study was performed of adult patients wait-listed for LT at LT centers in the United States between January 1, 2010, and December 31, 2017. Main outcomes and measures were (1) wait-listing rates by race/ethnicity in states that expanded Medicaid (expansion states) compared with those that did not (nonexpansion states) and (2) actual vs predicted rates of LT wait-listing by race/ethnicity after Medicaid expansion. The United Network for Organ Sharing (UNOS) maintains a database of all organ transplant data in the United States. These data include all candidates wait-listed for LT. This study was reviewed by an Indiana University School of Medicine institutional review board. It was deemed exempt from patient informed consent because deidentified data were used for the analysis. This study followed the Strengthening the Reporting of Observational Studies in Epidemiology (STROBE) reporting guideline.

The first round of Medicaid expansion occurred on January 1, 2014. Therefore, the post–Medicaid expansion era was defined as the period between January 1, 2014, and December 31, 2017. The pre–Medicaid expansion era was defined as the period between January 1, 2010, and December 31, 2013.

**Figure 1.**
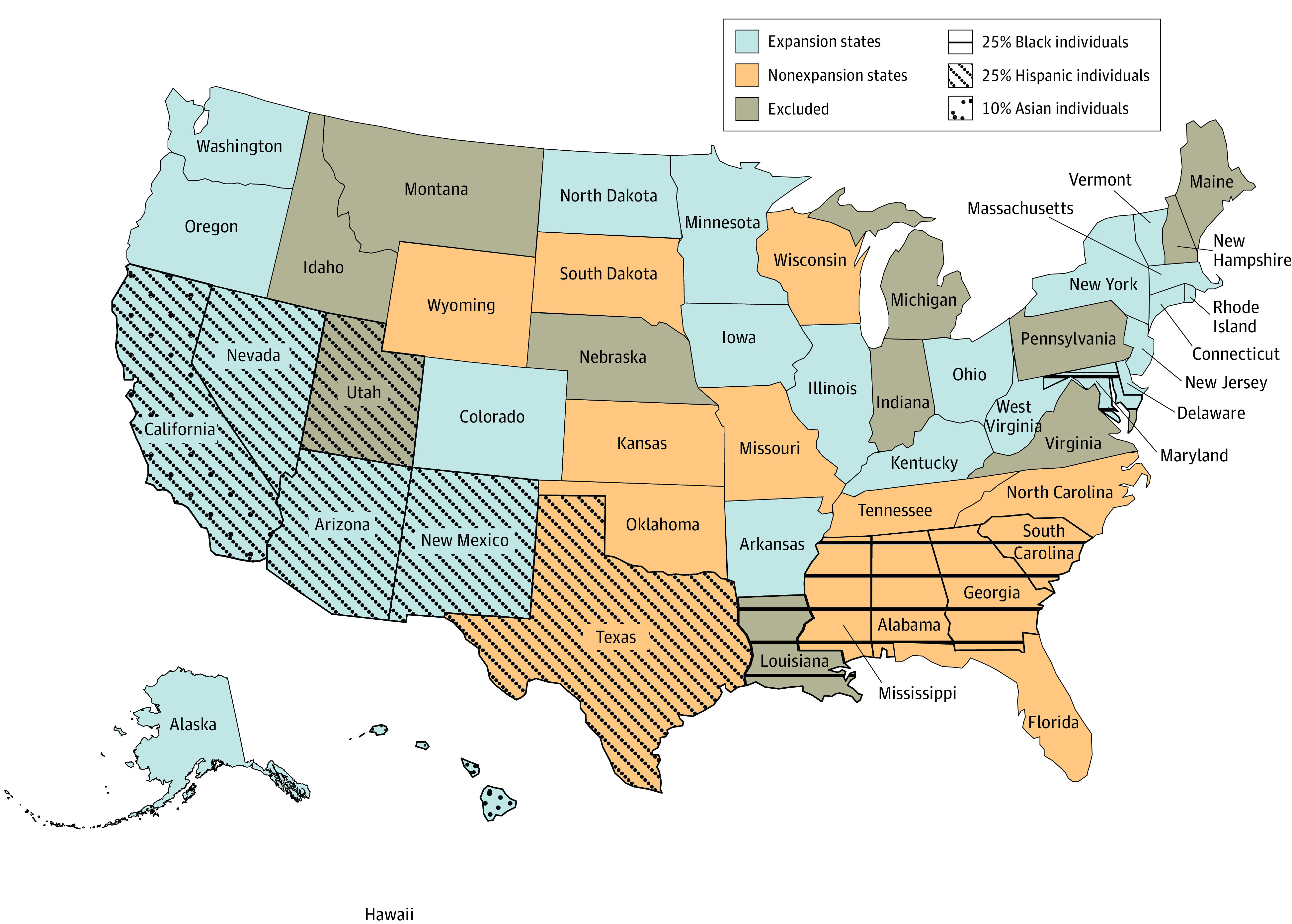
Populations of Racial/Ethnic Minorities in Expansion States and Nonexpansion States In 2014, the Affordable Care Act sought to expand insurance coverage through an optional state-level expansion of Medicaid. The map identifies states with higher populations of racial/ethnic minorities.

States that expanded Medicaid during the first round of expansion were called expansion states (Figure 1). States that never expanded were called nonexpansion states (Figure 1). States that expanded after January 1, 2014 (Idaho, Indiana, Louisiana, Maine, Montana, Nebraska, New Hampshire, Pennsylvania, Utah, and Virginia), were excluded from this analysis to allow for meaningful follow-up and the ability to assess trends with less confounding (Figure 1).

To account for changes in state population over time, a State Health Access Data Assistance Center population data set was obtained on July 8, 2019. The data were stratified by state, age, and race/ethnicity for 2010 to 2017. The University of Minnesota analyzes national population data from the American Community Survey and makes the data sets publicly available.^17^ After excluding states that expanded Medicaid after January 1, 2014, and residents younger than 18 years, the total adult population for expansion states and nonexpansion states was calculated for each year, stratified by race/ethnicity. This population was combined with data from the UNOS database to create a data set containing the number of individuals wait-listed with each insurance type within each year and each race/ethnicity.

### Covariates

Race/ethnicity was categorized as White, Black, Hispanic, or Asian based on UNOS classification. Patients of American Indian, Alaskan native, or Hawaiian/Pacific Islander descent and multiracial patients were categorized as other race/ethnicity.

The causes of liver disease were defined using UNOS primary diagnosis codes for acute liver failure from any cause, alcoholic liver disease (ALD) (alcoholic cirrhosis or acute alcoholic hepatitis), autoimmune liver disease, cholestatic liver diseases (primary biliary cholangitis, primary sclerosing cholangitis, or biliary cirrhosis), graft failure, hepatitis B virus (hepatitis B, hepatitis B, and hepatitis D), HCV (hepatitis C, hepatitis C and hepatitis B, or hepatitis C and alcoholic), nonalcoholic steatohepatitis (NASH), and other. Patients with a primary diagnosis code of NASH or cryptogenic cirrhosis who had a body mass index (calculated as weight in kilograms divided by height in meters squared) exceeding 30 were defined as having NASH. Insurance type was obtained from the UNOS database and defined as private, Medicaid, Medicare, or other.

### Statistical Analysis

Demographic and clinical variables for patients wait-listed in expansion states and nonexpansion states in the pre–Medicaid expansion and post–Medicaid expansion eras were described with summary statistics. The wait-listing rate for patients with ALD, HCV, or NASH was calculated for expansion states and nonexpansion states within each racial/ethnic group from 2010 to 2017.

The average incidence rate ratios (IRRs) comparing wait-listing rates in expansion states vs nonexpansion states were calculated before and after Medicaid expansion. The average IRR was calculated for the overall population and by race/ethnicity.

There are multiple factors that impact trends in LT wait-listing, and it is possible that trends were already occurring before Medicaid expansion. Therefore, to conjecture that a trend is associated with Medicaid expansion, the post–Medicaid expansion trajectory should be different than what would be predicted by the pre–Medicaid expansion trajectory. As such, segmented Poisson regression was used to conduct a controlled, interrupted time series analysis to compare actual vs predicted trend changes for LT wait-listing rates in expansion states compared with nonexpansion states. The Poisson regression was used to model trends in LT wait-listing rates in expansion states and nonexpansion states before and after Medicaid expansion for patients with private insurance, Medicaid, Medicare, or other insurance and overall for the full cohort, as well as by race/ethnicity. Both level and slope changes were allowed to account for immediate and gradual consequences of Medicaid expansion. From this model, annual percentage change (APC) in wait-listing rates adjusted for state population changes was estimated for expansion states and nonexpansion states. The IRRs comparing the observed wait-listing rate with what would have been observed if pre–Medicaid expansion trends had continued were also estimated for each year. The trend in those IRRs was further summarized by calculating the average APC.

Because Medicaid expansion occurred in 2014 at the same time as the market release of direct-acting antiviral agents (DAAs) for HCV, the previously mentioned analyses were also performed by excluding patients with HCV. The analyses were also performed excluding patients with hepatocellular carcinoma (HCC).

All analytic assumptions were tested and verified. Analyses were conducted in R, version 3.6.0 (R Core Team). Figures were produced using the ggplot2 package by Wickham.^18^ All tests were 2 sided, and *P* < .05 was considered statistically significant for all statistical tests.

## Results

Among 75 748 patients wait-listed for LT during the study period, the median age was 57.0 years (interquartile range [IQR], 50.0-62.0 years); 48 566 (64.1%) were male, and 27 182 (35.9%) were female. The proportion of Black individuals and Hispanic individuals in the cohort was 8.9% and 16.4%, respectively. The Black population and Hispanic population were greater than 25% in the following nonexpansion states: Alabama, Georgia, Mississippi, South Carolina, and Texas (Figure 1).

### Differences Between Patients Wait-listed Before vs After Medicaid Expansion

Patients were slightly younger in the pre–Medicaid expansion era compared with the post–Medicaid expansion era in expansion states (57.0 [IQR, 50.0-62.0] years vs 58.0 [IQR, 51.0-63.0] years) and nonexpansion states (median, 56.0 [IQR, 50.0-61.0] years vs 58.0 [IQR, 50.0-63.0] years) (Table). The number of Hispanic patients wait-listed for LT increased in the post–Medicaid expansion era in both expansion states and nonexpansion states (from 17.9% to 18.2% in expansion states and from 12.8% to 14.5% in nonexpansion states) (Table).

**Table.** Demographic and Clinical Characteristics of Patients Wait-listed for Liver Transplant in Expansion States and Nonexpansion States in the Pre–Medicaid Expansion Era and Post–Medicaid Expansion Era

Variable	No./total No. (%)				
	Expansion states		Nonexpansion states		Total (N = 75 748)
	Before Medicaid expansion (n = 23 017)	After Medicaid expansion (n = 23 858)	Before Medicaid expansion (n = 14 001)	After Medicaid expansion (n = 14 872)	
Age, median (IQR), y	57.0 (50.0-62.0)	58.0 (51.0-63.0)	56.0 (50.0-61.0)	58.0 (50.0-63.0)	57.0 (50.0-62.0)
Sex					
Male	15 078 (65.5)	15 247 (63.9)	8821 (63.0)	9420 (63.3)	48 566 (64.1)
Female	7939 (34.5)	8611 (36.1)	5180 (37.0)	5452 (36.7)	27 182 (35.9)
Race/ethnicity					
White	15 171 (65.9)	15 682 (65.7)	10 324 (73.7)	10 653 (71.6)	51 830 (68.4)
Black	1899 (8.3)	1940 (8.1)	1388 (9.9)	1488 (10.0)	6715 (8.9)
Hispanic	4128 (17.9)	4353 (18.2)	1792 (12.8)	2156 (14.5)	12 429 (16.4)
Asian	1495 (6.5)	1484 (6.2)	279 (2.0)	341 (2.3)	3599 (4.8)
Other	324 (1.4)	399 (1.7)	218 (1.6)	234 (1.6)	1175 (1.6)
Diagnosis					
Acute liver failure from any cause	1812 (7.9)	1400 (5.9)	982 (7.0)	759 (5.1)	4953 (6.5)
Alcoholic liver disease	3830 (16.6)	5615 (23.5)	2244 (16.0)	3340 (22.5)	15 029 (19.8)
Autoimmune liver disease	611 (2.7)	655 (2.7)	414 (3.0)	488 (3.3)	2168 (2.9)
Cholestatic liver diseases	1724 (7.5)	1809 (7.6)	1027 (7.3)	1027 (6.9)	5587 (7.4)
Graft failure	140 (0.6)	392 (1.6)	93 (0.7)	320 (2.2)	945 (1.2)
HBV	727 (3.2)	684 (2.9)	223 (1.6)	220 (1.5)	1854 (2.4)
HCV	9164 (39.8)	6987 (29.3)	5135 (36.7)	3856 (25.9)	25 142 (33.2)
NASH	2558 (11.1)	3926 (16.5)	1954 (14.0)	3004 (20.2)	11 442 (15.1)
Other	2451 (10.6)	2390 (10.0)	1929 (13.8)	1858 (12.5)	8628 (11.4)
Sodium level, median (IQR), mEq/L	137.0 (134.0-139.0)	137.0 (134.0-140.0)	137.0 (134.0-139.0)	137.0 (134.0-139.0)	137.0 (134.0-139.0)
MELD score, median (IQR)^a^	15.0 (11.0-22.0)	16.0 (11.0-25.0)	16.0 (12.0-22.0)	17.0 (12.0-25.0)	16.0 (11.0-24.0)
HCC	5871 (25.5)	7018 (29.4)	3041 (21.7)	3812 (25.6)	19 742 (26.1)
Dialysis	1692 (7.4)	2138 (9.0)	880 (6.3)	1259 (8.5)	5969 (7.9)
Diabetes	6102 (26.5)	6810 (28.5)	3818 (27.3)	4657 (31.3)	21 387 (28.2)
Educational attainment					
None	98/21 554 (0.5)	98/22 833 (0.4)	30/12 497 (0.2)	28/14 139 (0.2)	254/71 023 (0.4)
Grade school	1372/21 554 (6.4)	1532/22 833 (6.7)	555/12 497 (4.4)	645/14 139 (4.6)	4104/71 023 (5.8)
High school	9216/21 554 (42.8)	9462/22 833 (41.4)	5183/12 497 (41.5)	5829/14 139 (41.2)	29 690/71 023 (41.8)
Some college	5553/21 554 (25.8)	5765/22 833 (25.2)	3536/12 497 (28.3)	3829/14 139 (27.1)	18 683/71 023 (26.3)
Associate or bachelor’s degree	3568/21 554 (16.6)	4113/22 833 (18.0)	2400/12 497 (19.2)	2792/14 139 (19.7)	12 873/71 023 (18.1)
Graduate degree	1747/21 554 (8.1)	1863/22 833 (8.2)	793/12 497 (6.3)	1016/14 139 (7.2)	5419/71 023 (7.6)
Working for income	4867 (21.1)	5266 (22.1)	2996 (21.4)	3236 (21.8)	16 365 (21.6)

Abbreviations: HBV, hepatitis B virus; HCC, hepatocellular carcinoma; HCV, hepatitis C virus; IQR, interquartile range; MELD, Model for End-Stage Liver Disease; NASH, nonalcoholic steatohepatitis.

SI conversion factor: To convert sodium level to millimoles per liter, multiply by 1.0.

^a^ The MELD score is used to assess liver disease severity, and high MELD scores indicate worse liver function.

The proportion of patients wait-listed with ALD or NASH was higher in the post–Medicaid expansion era in expansion states and nonexpansion states (Table and eFigure 1 in the Supplement). The proportion of patients wait-listed with HCV was lower in the post–Medicaid expansion era in expansion states and nonexpansion states (Table and eFigure 1 in the Supplement). The proportion of patients with HCC increased in the post–Medicaid expansion era in expansion states and nonexpansion states.

The proportion of patients wait-listed and working for income in the post–Medicaid expansion era was similar in expansion states (22.1%) and nonexpansion states (21.8%). Educational attainment was also similar in the post–Medicaid expansion era for patients in expansion states and nonexpansion states (41.4% with high school completion after Medicaid expansion in expansion states and 41.2% with high school completion in nonexpansion states) (Table).

### Differences in Wait-listing Rates of Racial/Ethnic Minorities Between Expansion States vs Nonexpansion States

**Figure 2.**
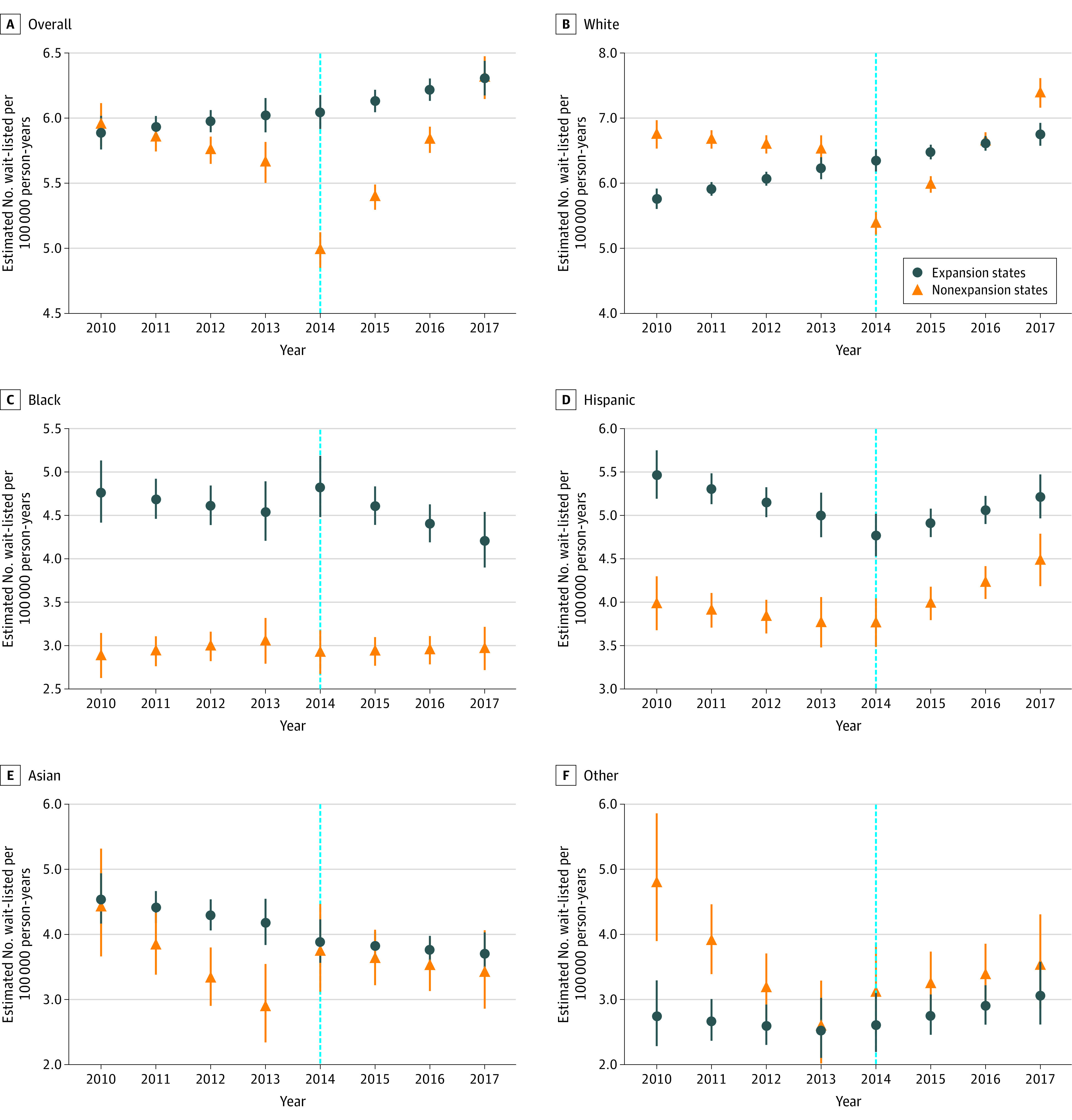
Rate of Patients Being Wait-listed Each Year in Expansion States and Nonexpansion States A-F, Patients of American Indian, Alaskan native, or Hawaiian/Pacific Islander descent and multiracial patients were categorized as other race/ethnicity. The vertical lines on either side of the data markers represent the 95% CIs. The dotted light blue line indicates the year when Medicaid expansion occurred.

**Figure 3.**
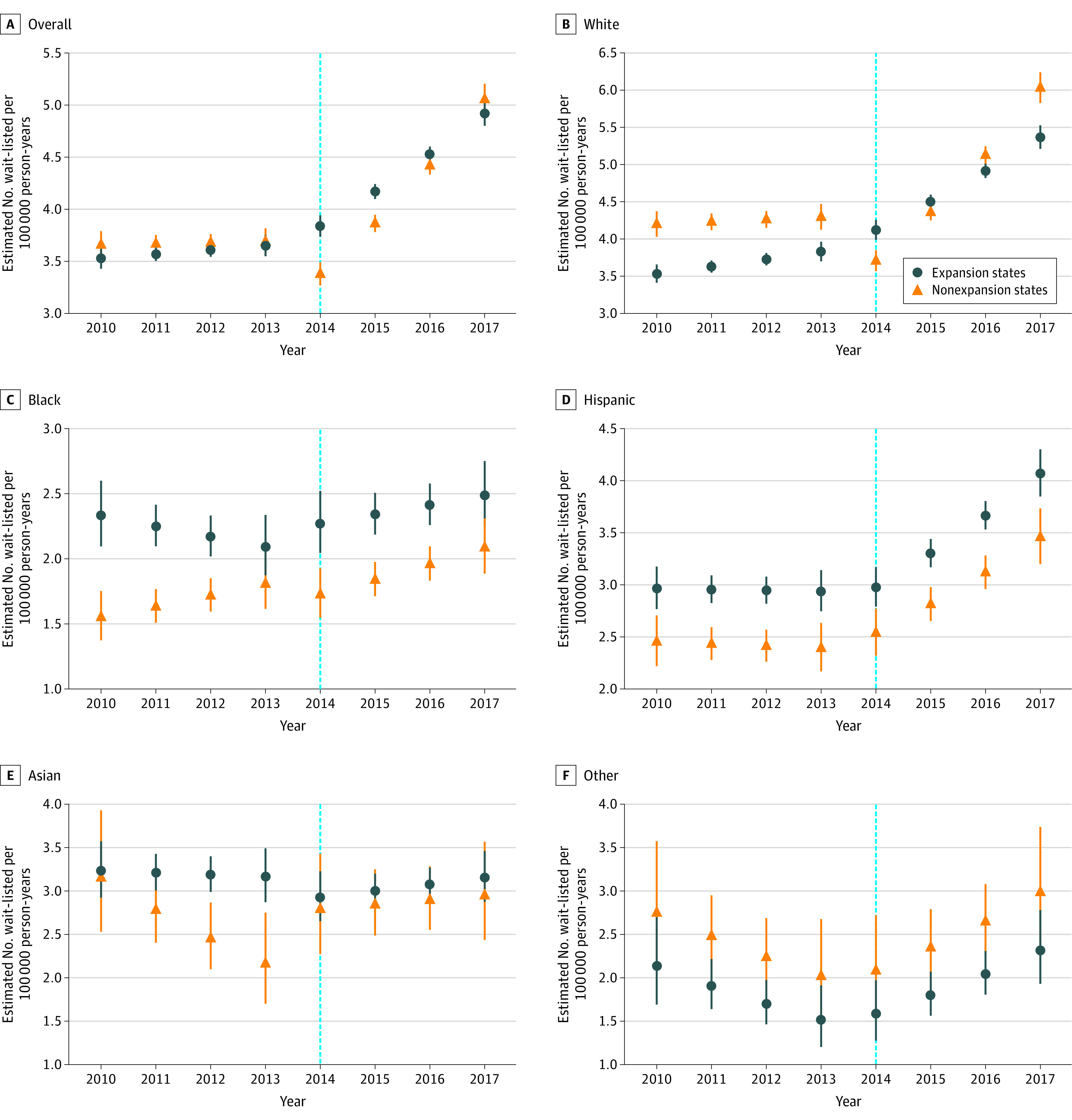
Rate of Patients Being Wait-listed Each Year in Expansion States and Nonexpansion States, Excluding Patients With Hepatitis C Virus A-F, Patients of American Indian, Alaskan native, or Hawaiian/Pacific Islander descent and multiracial patients were categorized as other race/ethnicity. The vertical lines on either side of the data markers represent the 95% CIs. The dotted light blue line indicates the year when Medicaid expansion occurred.

When controlling for state population, more patients have been wait-listed for LT in expansion states compared with nonexpansion states since 2012 (Figure 2A), contributing to higher rates of wait-listing of Black patients and Hispanic patients in expansion states (Figure 2C and D). Black patients and Hispanic patients were statistically significantly more likely to be wait-listed in expansion states than in nonexpansion states (IRR, 1.54 [95% CI, 1.44-1.64] for Black patients and 1.21 [95% CI, 1.15-1.28] for Hispanic patients) (Figure 2C and D and eTable 1 in the Supplement), which remained true when patients with HCV were excluded from the analysis (Figure 3C and D and eTable 2 in the Supplement).

### Differences Between Wait-listing Rates of Racial/Ethnic Minorities Before vs After Medicaid Expansion

Overall, wait-listing rates increased with time in expansion states and nonexpansion states in the post–Medicaid expansion era (APC, 1.4% [95% CI, 0.3%-2.6%] for expansion states and 8.2% [95% CI, 6.6%-9.7%] for nonexpansion states) (Figure 2A and eTable 3 in the Supplement). Specifically, in the post–Medicaid expansion era, wait-listing rates increased with time in White patients and Hispanic patients in both expansion states and nonexpansion states (Figure 2B and D and eTable 3 in the Supplement). Among Black patients in the post–Medicaid expansion era, the wait-listing rate decreased with time in expansion states (APC, −4.4%; 95% CI, −8.2% to −0.6%) but not in nonexpansion states (APC, 0.5%; 95% CI, −4.0% to 5.2%) (Figure 2C and eTable 3 in the Supplement).

The market release of DAAs for HCV occurred in 2014, the same year as Medicaid expansion. When Black patients with HCV were excluded from the analysis, the wait-listing rates for Black patients in expansion states no longer decreased and was stable (APC, 3.1%; 95% CI, −2.4% to 8.9%) (Figure 3C and eTable 4 in the Supplement). The post–Medicaid expansion trends were similar in White patients and Hispanic patients when patients with HCV were excluded (Figure 3B and D and eTable 4 in the Supplement).

### Contributions of Medicaid Expansion to Differences in Wait-listing Rates for Racial/Ethnic Minorities

**Figure 4.**
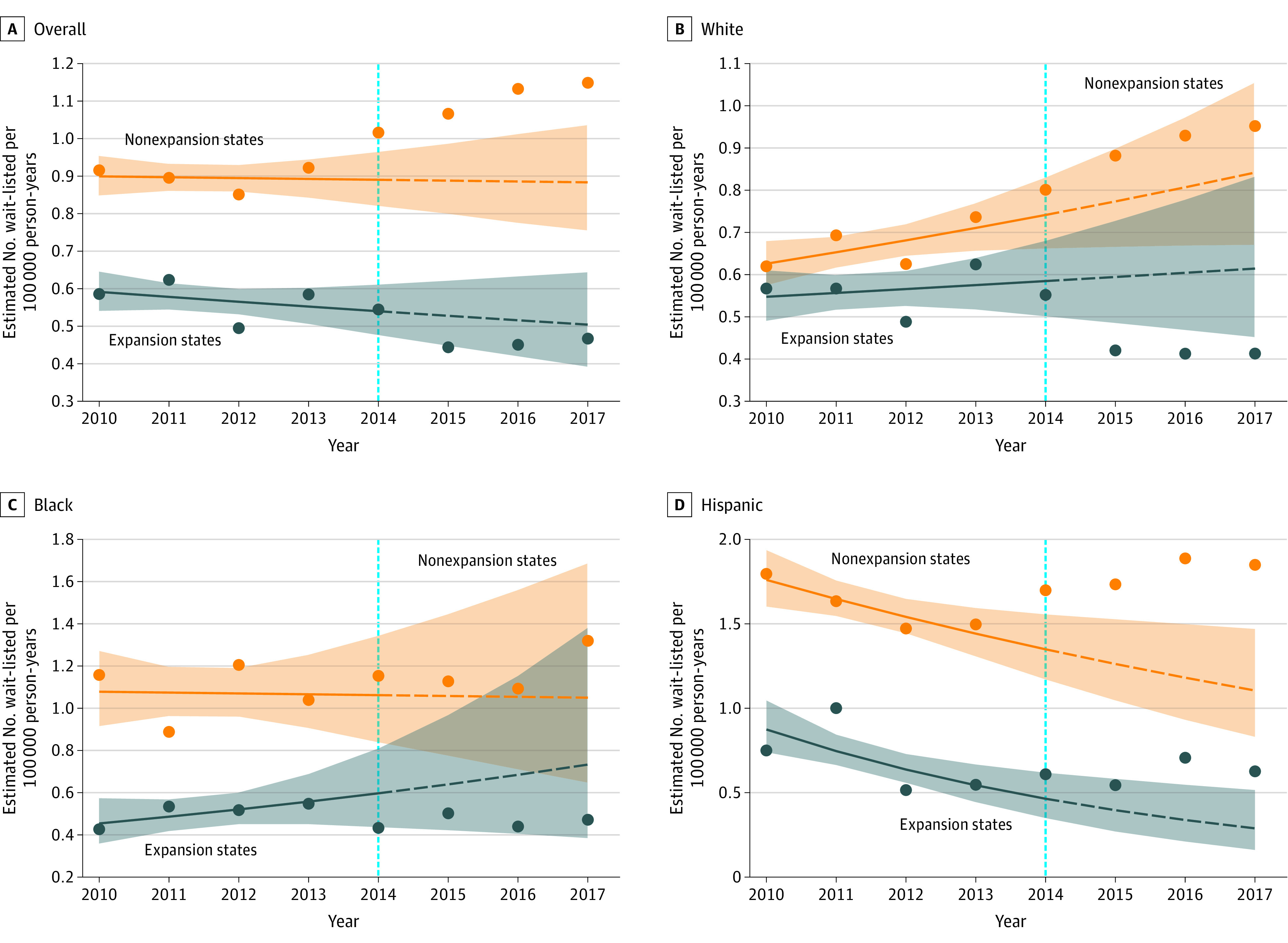
Actual vs Predicted Rates of Medicaid Patients Being Wait-listed for Liver Transplant in Expansion States vs Nonexpansion States A-D, The implications of Medicaid expansion for wait-listing trends in racial/ethnic minorities is shown. The shaded areas represent the 95% CIs. The dotted light blue line indicates the year when Medicaid expansion occurred.

Given that wait-listing rates increased over time in the post–Medicaid expansion era among White patients and Hispanic patients in expansion states, we sought to explore if this increase could be attributed to Medicaid expansion. The actual rate of wait-listing of White Medicaid patients in expansion states was not more than would have been predicted by pre–Medicaid expansion trends (APC, 1.4%; 95% CI, −3.7% to 6.8%) (Figure 4B and eTable 5 and eTable 7 in the Supplement). In White patients, the increased wait-listing could be attributed to higher rates of wait-listing patients with private insurance than would have been predicted without Medicaid expansion (APC, 3.3%; 95% CI, 0.6%-5.2%) (eFigure 2, eTable 5, and eTable 7 in the Supplement). Hispanic Medicaid patients were wait-listed at higher rates in the post–Medicaid expansion era than would have been predicted without Medicaid expansion, but this finding was true in expansion states and nonexpansion states (APC, 10.6% [95% CI, 3.4%-18.2%] in expansion states; 21.2% [95% CI, 5.5%-39.1%] in nonexpansion states) (Figure 4D and eTable 5 and eTable 7 in the Supplement). Black Medicaid patients in expansion states were wait-listed at the same rate in the post–Medicaid expansion era as would be predicted by pre–Medicaid expansion trends (APC, 4.5%; 95% CI, −6.8% to 17.2%) (Figure 4C and eTable 5 and eTable 7 in the Supplement).

When patients with HCV were excluded from the analysis, Hispanic Medicaid patients were statistically significantly more likely to be wait-listed in the post–Medicaid expansion era than would have been predicted without Medicaid expansion (APC, 13.2%; 95% CI, 4.0%-23.2%) (eFigure 3, eTable 6, and eTable 8 in the Supplement). In contrast, White Medicaid patients without HCV were wait-listed at lower rates than would have been predicted in nonexpansion states (eFigure 3, eTable 6, and eTable 8 in the Supplement).

This cohort included patients with HCC. To ensure that inclusion of this population of patients did not change the results, each analysis was performed after excluding this group. There was no statistically significant change in the magnitude or direction of the findings.

## Discussion

Although the full burden of decompensated liver disease necessitating LT is unknown, data suggest that there are fewer racial/ethnic minorities wait-listed for LT than one would predict based on their burden of liver disease.^1,2^ One of the approaches to reducing racial/ethnic disparities in health care has been to expand insurance coverage.^19^ However, the path to LT is complex. It has been unclear how expanding insurance coverage might impact LT wait-listing trends for racial/ethnic minorities. Our data indicate that where you live may be as important as insurance coverage because statistically significantly more Black patients and Hispanic patients were wait-listed in expansion states compared with nonexpansion states even before Medicaid expansion. We further demonstrate that the wait-listing rates for Black patients with HCV is downtrending in expansion states but not nonexpansion states. Finally, Medicaid expansion was associated with a higher rate of wait-listing of Hispanic patients without HCV. Although these data are observational, they suggest an important association between Medicaid expansion and LT wait-listing rates in Black patients and Hispanic patients with ESLD.

Black patients and Hispanic patients were between 21% and 54% more likely to be wait-listed in expansion states compared with nonexpansion states. One might hypothesize that the burden of HCV varies by race/ethnicity and by state, contributing to higher rates of wait-listing of racial/ethnic minorities in expansion states. However, when patients with HCV were excluded from the analysis, these differences remained. Geographic differences in access to LT have long been described and were the premise behind the Institute of Medicine’s mandate to the LT community to develop an allocation system that ensured equality in access to LT.^20^ Differences in LT center wait-listing behavior are complex and subject to multiple factors, including competition in the area, regulatory pressures, practice patterns, and local organ availability.^1^ Although these differences might explain state and regional variations in wait-listing, it is not clear that they explain geographic differences in wait-listing for racial/ethnic minorities.

We found that LT wait-listing rates increased in the post–Medicaid expansion era for White patients and Hispanic patients in expansion states and nonexpansion states. The increase seen among White patients was associated with higher wait-listing rates of White patients with private insurance than would have been predicted without Medicaid expansion. In the full cohort of Hispanic patients, there were increased wait-listing rates seen in both expansion states and nonexpansion states, making it difficult to attribute the increased wait-listing rates to Medicaid expansion alone. However, when patients with HCV were excluded from the analysis, statistically significantly more Hispanic patients with non–HCV-related ESLD were wait-listed in expansion states than would have been predicted if Medicaid expansion had not occurred. This observation was not seen in nonexpansion states, suggesting an association between Medicaid expansion and wait-listing rates of Hispanic patients with non–HCV-related ESLD. Previous work in this area has yielded mixed results. Two studies^15,16^ examining solid organ transplants found increases in the proportion of Medicaid patients wait-listed for LT in expansion states after Medicaid expansion; however, there was no information provided about the race/ethnicity of the population. A final study^14^ that included the race/ethnicity of LT candidates found no difference in the number of patients wait-listed by race/ethnicity after Medicaid expansion. However, that study was limited to 1 year of data. Those previous studies also did not account for wait-listing trends that were already under way before Medicaid expansion or explore trends in other insurance types. Because these policies particularly impact racial/ethnic minorities, such nuances are relevant and important.

Wait-listing rates decreased for Black patients in expansion states; however, when patients with HCV were excluded, there was a non–statistically significant change in wait-listing rates. This finding would suggest that the decreased wait-listing rate seen in Black patients in expansion states contributed to lower rates of wait-listing in patients with HCV. This trend was not seen in nonexpansion states, suggesting that the downward trend seen in Black patients was not secondary to the market release of DAAs and HCV treatment alone. Such trends may be associated with increased access to DAA therapy in expansion states secondary to Medicaid expansion. Medicaid requirements for DAA therapy eligibility vary by state; however, there have been state-based initiatives to increase access to therapy for as many Medicaid patients with chronic HCV as possible.^21^

### Strengths and Limitations

A strength of our study is its large sample size that includes a diverse population of patients with ESLD. This population is also the most contemporary such cohort examined, including multiple years of post–Medicaid expansion data. Given the complex landscape of LT allocation, we also used methods to attempt to account for trends that may have already been in process before Medicaid expansion. We explored non-Medicaid insurance types to identify their contribution to overall trends. Finally, we controlled for changes in state populations so that differences in trends between expansion states and nonexpansion states could be better compared.

Our study also has limitations. There are no clear data on the number of patients with each cause of ESLD in every state, so we could not control for both state population and each cause of disease individually. However, exclusion of patients with HCV allowed exploration of important questions about the impact of the market release of DAAs. The ACA included policies beyond just Medicaid expansion, and it is possible that these changes also impacted the results of the present study. Finally, we were unable to explore the reasons why Black patients and Hispanic patients were statistically significantly more likely to be wait-listed in expansion states vs nonexpansion states.

## Conclusions

There are 2 important observations of our study. First, where a patient lives could impact the likelihood of LT wait-listing for racial/ethnic minority patients. Second, Medicaid expansion may be associated with LT wait-listing trends for Black patients and Hispanic patients. In the current political climate in which changes to health care policy and coverage are being regularly debated, it is important to understand the potential impact that expanded coverage has had for vulnerable populations. Future studies should explore geographic and other barriers to LT referral and wait-listing.

## Notes

Supplement. **eFigure 1.** Rate of Patients Being Waitlisted With Hepatitis C Virus, Non-alcoholic Steatohepatitis, and Alcoholic Liver Disease in Expand and Non-expand States **eFigure 2.** Actual Versus Predicted Number of Privately Insured Patients Being Waitlisted for Liver Transplantation **eFigure 3.** Actual vs Predicted Number of Medicaid Patients Without Hepatitis C Virus Being Waitlisted for Liver Transplantation **eTable 1.** Estimated Incidence Rate Ratios Comparing Waitlisting Rates in Expand vs Non-expand States Pre- and Post-Medicaid Expansion **eTable 2.** Estimated Incidence Rate Ratios Comparing Waitlisting Rates in Expand vs Non-expand States in Patients Without Hepatitis C Virus **eTable 3.** Estimated Annual Percent Change in Number Waitlisted per 100,000 Person-Years Pre- and Post-Medicaid Expansion in Expand and Non-expand States **eTable 4.** Estimated Annual Percent Change in Number Waitlisted per 100,000 Person-Years Pre- and Post-Medicaid Expansion in Expand and Non-expand States in Patients Without Hepatitis C Virus **eTable 5.** Estimated Annual Percent Change in the Incidence Rate Ratio Comparing Observed vs Expected Waitlisting Rates in Expand and Non-expand States **eTable 6.** Estimated Annual Percent Change in the Incidence Rate Ratio Comparing Observed vs Expected Waitlisting Rates in Expand and Non-expand States in Patients Without Hepatitis C Virus **eTable 7.** Estimated Incidence Rate Ratios Comparing Post-Medicaid Expansion Waitlisting Rates to What Would Have Been Observed if the Pre-Medicaid Expansion Era Trends Had Continued in Expand and Non-expand States **eTable 8.** Estimated Incidence Rate Ratios Comparing Post-Medicaid Expansion Waitlisting Rates to What Would Have Been Observed if the Pre-Medicaid Expansion Era Trends Had Continued in Expand and Non-expand States in Patients Without Hepatitis C Virus
