# Supplementary material for: Association of State Medicaid Expansion With Racial/Ethnic Disparities in Liver Transplant Wait-listing in the United States
Source: JAMA Netw Open. 2020 Oct 8;3(10):e2019869. doi: 10.1001/jamanetworkopen.2020.19869 (PMC7545310; doi:10.1001/jamanetworkopen.2020.19869)
Supplement: Supplement. — eFigure 1. Rate of Patients Being Waitlisted With Hepatitis C Virus, Non-alcoholic Steatohepatitis, and Alcoholic Liver Disease in Expand and Non-expand States eFigure 2. Actual Versus Predicted Number of Privately Insured Patients Being Waitlisted for Liver Transplantation eFigure 3. Actual vs Predicted Number of Medicaid Patients Without Hepatitis C Virus Being Waitlisted for Liver Transplantation eTable 1. Estimated Incidence Rate Ratios Comparing Waitlisting Rates in Expand vs Non-expand States Pre- and Post-Medicaid Expansion eTable 2. Estimated Incidence Rate Ratios Comparing Waitlisting Rates in Expand vs Non-expand States in Patients Without Hepatitis C Virus eTable 3. Estimated Annual Percent Change in Number Waitlisted per 100,000 Person-Years Pre- and Post-Medicaid Expansion in Expand and Non-expand States eTable 4. Estimated Annual Percent Change in Number Waitlisted per 100,000 Person-Years Pre- and Post-Medicaid Expansion in Expand and Non-expand States in Patients Without Hepatitis C Virus eTable 5. Estimated Annual Percent Change in the Incidence Rate Ratio Comparing Observed vs Expected Waitlisting Rates in Expand and Non-expand States eTable 6. Estimated Annual Percent Change in the Incidence Rate Ratio Comparing Observed vs Expected Waitlisting Rates in Expand and Non-expand States in Patients Without Hepatitis C Virus eTable 7. Estimated Incidence Rate Ratios Comparing Post-Medicaid Expansion Waitlisting Rates to What Would Have Been Observed if the Pre-Medicaid Expansion Era Trends Had Continued in Expand and Non-expand States eTable 8. Estimated Incidence Rate Ratios Comparing Post-Medicaid Expansion Waitlisting Rates to What Would Have Been Observed if the Pre-Medicaid Expansion Era Trends Had Continued in Expand and Non-expand States in Patients Without Hepatitis C Virus [file jamanetwopen-e2019869-s001.pdf]

Supplementary Online Content

Nephew LD, Mosesso K, Desai A, et al. Association of state Medicaid expansion with racial/ethnic disparities in liver transplant wait-listing in the United States. *JAMA Netw Open*. 2020;3(10):e2019869. doi:10.1001/jamanetworkopen.2020.19869

eFigure 1. Rate of Patients Being Waitlisted With Hepatitis C Virus, Non-alcoholic Steatohepatitis, and Alcoholic Liver Disease in Expand and Non-expand States

eFigure 2. Actual vs Predicted Number of Privately Insured Patients Being Waitlisted for Liver Transplantation

eFigure 3. Actual vs Predicted Number of Medicaid Patients Without Hepatitis C Virus Being Waitlisted for Liver Transplantation

eTable 1. Estimated Incidence Rate Ratios Comparing Waitlisting Rates in Expand vs Non-expand States Pre- and Post-Medicaid Expansion

eTable 2. Estimated Incidence Rate Ratios Comparing Waitlisting Rates in Expand vs Non-expand States in Patients Without Hepatitis C Virus

eTable 3. Estimated Annual Percent Change in Number Waitlisted per 100,000 Person-Years Pre- and Post-Medicaid Expansion in Expand and Non-expand States

eTable 4. Estimated Annual Percent Change in Number Waitlisted per 100,000 Person-Years Pre- and Post-Medicaid Expansion in Expand and Non-expand States in Patients Without Hepatitis C Virus

eTable 5. Estimated Annual Percent Change in the Incidence Rate Ratio Comparing Observed vs Expected Waitlisting Rates in Expand and Non-expand States

eTable 6. Estimated Annual Percent Change in the Incidence Rate Ratio Comparing Observed vs Expected Waitlisting Rates in Expand and Non-expand States in Patients Without Hepatitis C Virus

eTable 7. Estimated Incidence Rate Ratios Comparing Post-Medicaid Expansion Waitlisting Rates to What Would Have Been Observed if the Pre-Medicaid Expansion Era Trends Had Continued in Expand and Non-expand States

eTable 8. Estimated Incidence Rate Ratios Comparing Post-Medicaid Expansion Waitlisting Rates to What Would Have Been Observed if the Pre-Medicaid Expansion Era Trends Had Continued in Expand and Non-expand States in Patients Without Hepatitis C Virus

This supplementary material has been provided by the authors to give readers additional information about their work.

© Nephew LD et al. *JAMA Network Open*.

eFigure 1. Rate of patients being waitlisted with hepatitis C virus, non-alcoholic steatohepatitis, and alcoholic liver disease in expand and non-expand states.
Panel A Hepatitis C Virus (HCV) in non-expand and expand states, Panel B Alcoholic Liver Disease in non-expand and expand states, Panel C Non-alcoholic steatohepatitis (NASH) in non-expand and expand states.

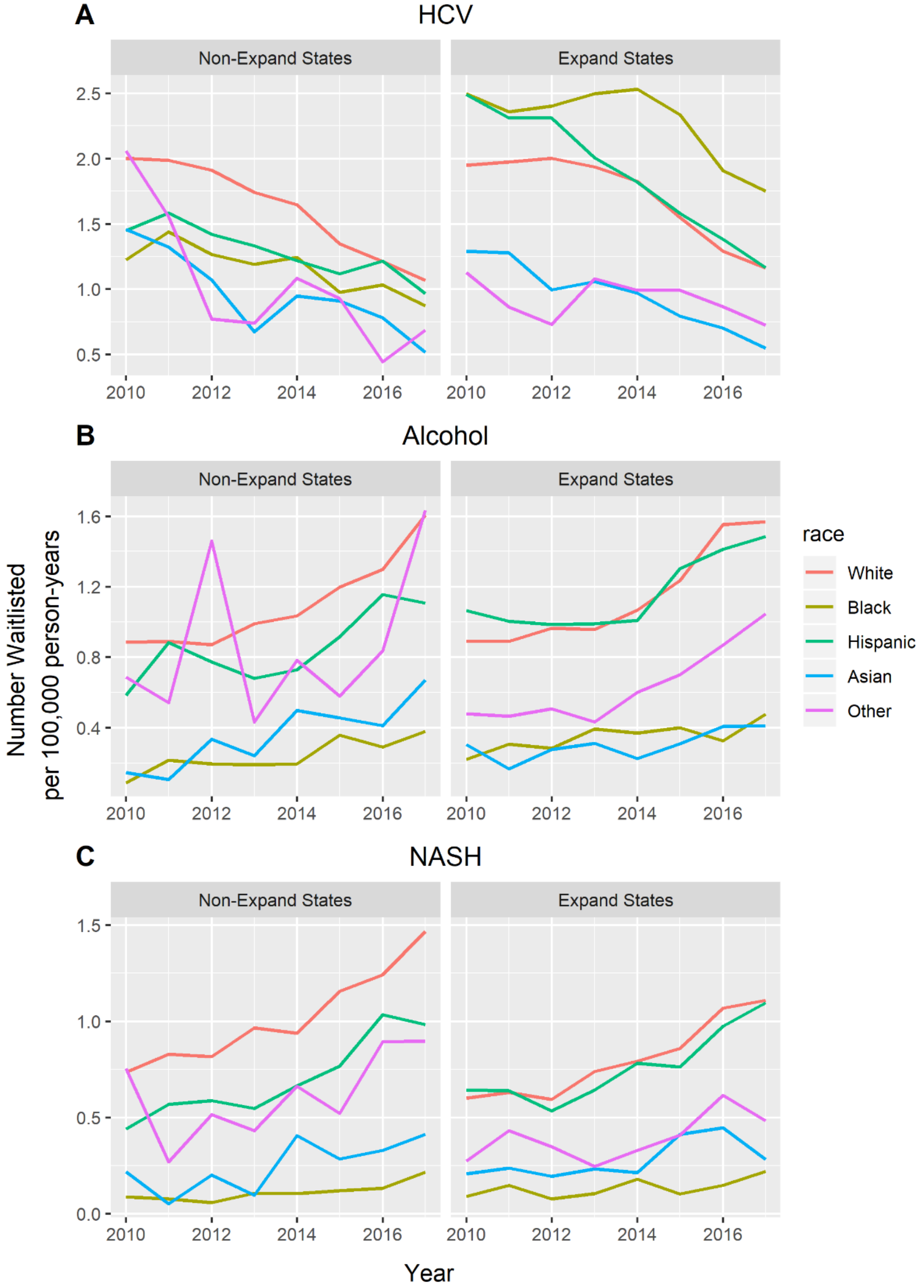

© Nephew LD et al. *JAMA Network Open*.

eFigure 2. Actual versus predicted number of privately insured patients being waitlisted for liver transplantation

Panel A Overall, Panel B White, Panel C Black, Panel D Hispanic

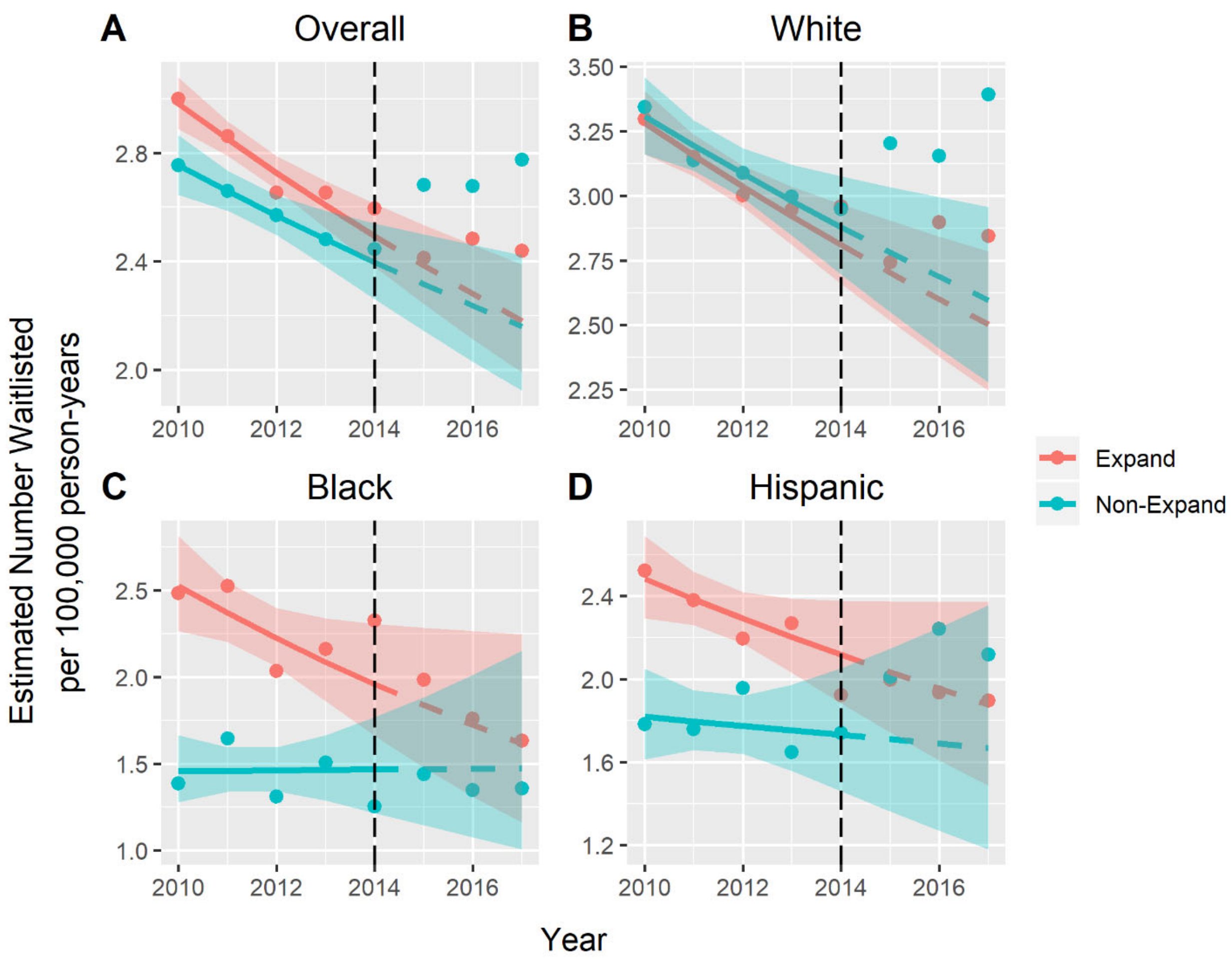

© Nephew LD et al. *JAMA Network Open*.

eFigure 3. Actual versus predicted number of Medicaid patients without hepatitis-C virus being waitlisted for liver transplantation
Panel A Overall, Panel B White, Panel C Black, Panel D Hispanic

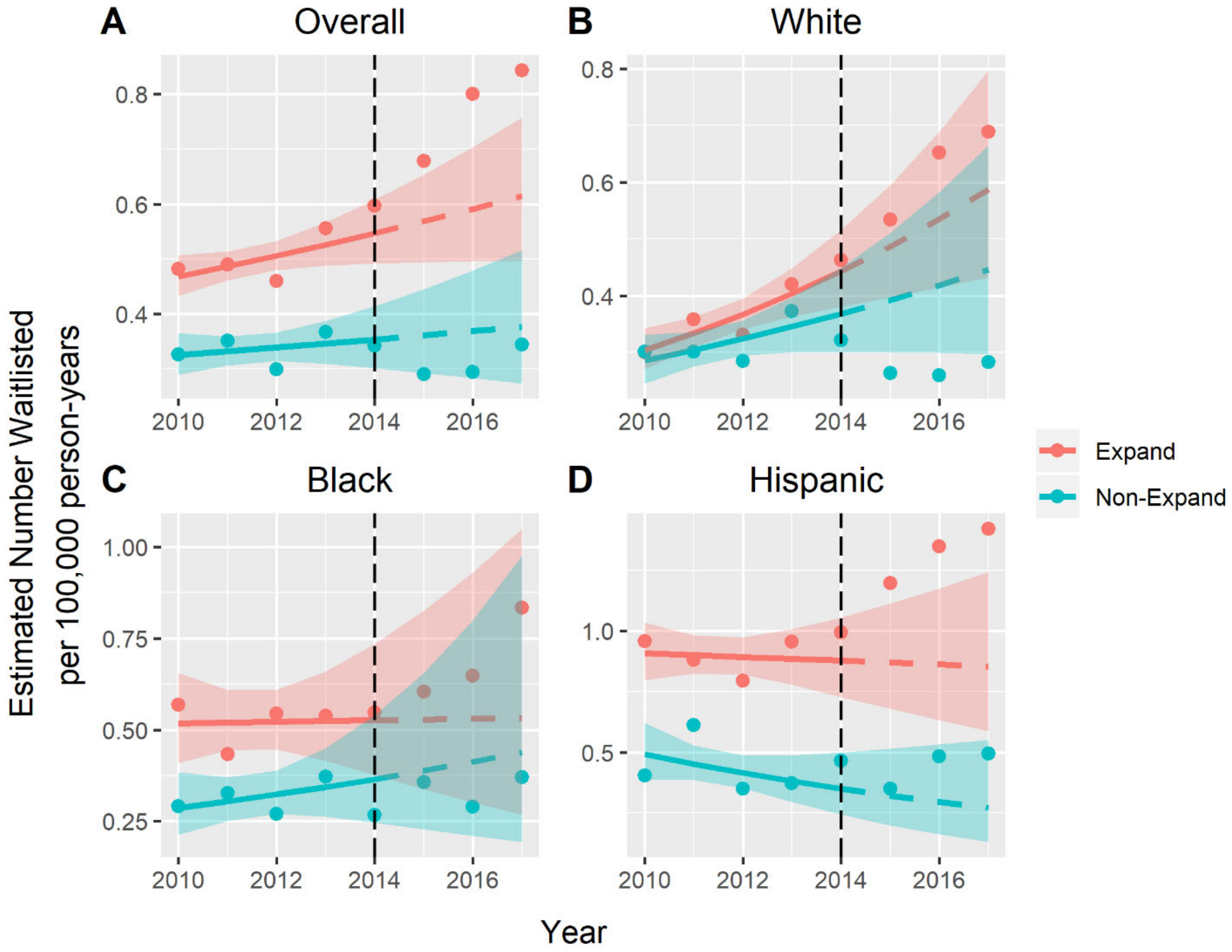

© Nephew LD et al. *JAMA Network Open*.

eTable 1. Estimated incidence rate ratios comparing waitlisting rates in expand vs non-expand states pre- and post-Medicaid expansion

Race	Pre-Medicaid	Post-Medicaid
Overall	1.03 (1.01, 1.05)	1.10 (1.08, 1.12)
White	0.90 (0.88, 0.93)	1.04 (1.01, 1.07)
Black	1.57 (1.47, 1.68)	1.54 (1.44, 1.64)
Hispanic	1.35 (1.28, 1.43)	1.21 (1.15, 1.28)
Asian	1.21 (1.07, 1.38)	1.06 (0.95, 1.20)
Other	0.74 (0.62, 0.87)	0.86 (0.73, 1.01)

eTable 2. Estimated incidence rate ratios comparing waitlisting rates in expand vs non-expand states in patients without hepatitis-C virus

Race	Pre-Medicaid	Post-Medicaid
Overall	0.98 (0.95, 1.00)	1.05 (1.03, 1.08)
White	0.87 (0.84, 0.89)	1.00 (0.97, 1.02)
Black	1.32 (1.20, 1.45)	1.25 (1.14, 1.36)
Hispanic	1.22 (1.14, 1.31)	1.17 (1.10, 1.25)
Asian	1.22 (1.05, 1.42)	1.06 (0.93, 1.21)
Other	0.76 (0.61, 0.94)	0.77 (0.64, 0.93)

© Nephew LD et al. *JAMA Network Open*.

eTable 3. Estimated annual percent change in number waitlisted per 100,000 person-years pre- and post-Medicaid expansion in expand and non-expand states

		**Expand States**		**Non-Expand States**	
Insurance	Race	pre-Medicaid	post-Medicaid	pre-Medicaid	post-Medicaid
Overall	Overall	0.8% (-0.4%, 1.9%)	**1.4% (0.3%, 2.6%)**	**-1.7% (-3.1%, -0.2%)**	**8.2% (6.6%, 9.7%)**
	White	**2.6% (1.2%, 4.1%)**	**2.1% (0.6%, 3.5%)**	-1.1% (-2.8%, 0.6%)	**11.1% (9.3%, 13.0%)**
	Black	-1.6% (-5.5%, 2.4%)	**-4.4% (-8.2%, -0.6%)**	1.9% (-2.8%, 6.8%)	0.5% (-4.0%, 5.2%)
	Hispanic	**-2.9% (-5.5%, -0.2%)**	**3.0% (0.3%, 5.8%)**	-1.9% (-5.8%, 2.3%)	**6.0% (2.1%, 10.1%)**
	Asian	-2.7% (-7.0%, 1.8%)	-1.6% (-5.9%, 3.0%)	**-13.3% (-21.9%, -3.6%)**	-3.0% (-11.8%, 6.7%)
	Other	-2.8% (-11.8%, 7.2%)	5.5% (-3.4%, 15.2%)	**-18.6% (-27.8%, -8.2%)**	4.3% (-7.0%, 16.9%)

eTable 4. Estimated annual percent change in number waitlisted per 100,000 person-years pre- and post-Medicaid expansion in expand and non-expand states in patients without hepatitis-C virus

		**Expand States**		**Non-Expand States**	
Insurance	Race	pre-Medicaid	post-Medicaid	pre-Medicaid	post-Medicaid
Overall	Overall	1.1% (-0.4%, 2.7%)	**8.6% (7.2%, 10.1%)**	0.3% (-1.6%, 2.2%)	**14.4% (12.5%, 16.3%)**
	White	**2.7% (0.8%, 4.7%)**	**9.2% (7.4%, 11.0%)**	0.8% (-1.4%, 3.0%)	**17.6% (15.4%, 19.9%)**
	Black	-3.6% (-9.0%, 2.2%)	3.1% (-2.4%, 8.9%)	5.2% (-1.2%, 12.0%)	**6.5% (0.7%, 12.7%)**
	Hispanic	-0.3% (-3.9%, 3.4%)	**11.0% (7.5%, 14.6%)**	-0.8% (-5.9%, 4.5%)	**10.9% (6.0%, 15.9%)**
	Asian	-0.7% (-5.8%, 4.7%)	2.5% (-2.5%, 7.9%)	**-11.8% (-22.0%, -0.2%)**	1.8% (-8.4%, 13.2%)
	Other	-10.8% (-21.3%, 1.1%)	**13.5% (2.0%, 26.3%)**	-9.8% (-22.0%, 4.4%)	12.8% (-1.2%, 28.7%)

© Nephew LD et al. *JAMA Network Open*.

eTable 5. Estimated annual percent change in the incidence rate ratio comparing observed vs expected waitlisting rates in expand and non-expand states

		**Expand States**	**Non-Expand States**
Insurance	Race	Annual Percent Change	Annual Percent Change
Overall	Overall	0.7% (-1.0%, 2.3%)	**10.0% (7.8%, 12.3%)**
	White	-0.6% (-2.5%, 1.5%)	**12.4% (9.7%, 15.1%)**
	Black	-2.9% (-8.2%, 2.8%)	-1.4% (-7.7%, 5.3%)
	Hispanic	**6.1% (2.2%, 10.2%)**	**8.0% (2.1%, 14.3%)**
	Asian	1.2% (-5.1%, 7.9%)	11.8% (-2.9%, 28.9%)
	Other	8.5% (-4.9%, 23.7%)	**28.1% (8.5%, 51.2%)**
Private	Overall	**2.9% (0.6%, 5.2%)**	**7.4% (4.5%, 10.4%)**
	White	**3.3% (0.6%, 6.0%)**	**7.8% (4.4%, 11.2%)**
	Black	-5.5% (-13.1%, 2.8%)	1.5% (-7.7%, 11.5%)
	Hispanic	3.3% (-2.6%, 9.6%)	8.2% (-0.3%, 17.3%)
	Asian	7.1% (-2.1%, 17.2%)	8.8% (-8.8%, 29.8%)
	Other	9.0% (-10.6%, 33.0%)	20.3% (-5.3%, 52.8%)
Medicaid	Overall	**4.6% (0.9%, 8.5%)**	-2.4% (-8.2%, 3.8%)
	White	1.4% (-3.7%, 6.8%)	**-10.4% (-17.2%, -3.0%)**
	Black	4.5% (-6.8%, 17.2%)	-5.3% (-19.5%, 11.3%)
	Hispanic	**10.6% (3.4%, 18.2%)**	**21.2% (5.5%, 39.1%)**
	Asian	-2.0% (-13.5%, 11.0%)	43.3% (-12.1%, 133.6%)
	Other	26.4% (-0.7%, 60.9%)	23.0% (-15.6%, 79.1%)

© Nephew LD et al. *JAMA Network Open*.

eTable 6. Estimated annual percent change in the incidence rate ratio comparing observed vs expected waitlisting rates in expand and non-expand states. in patients without hepatitis-c virus

		**Expand States**	**Non-Expand States**
Insurance	Race	Annual Percent Change	Annual Percent Change
Overall	Overall	**7.4% (5.2%, 9.6%)**	**14.1% (11.3%, 17.0%)**
	White	**6.3% (3.7%, 9.0%)**	**16.8% (13.4%, 20.2%)**
	Black	6.9% (-1.3%, 15.8%)	1.2% (-7.0%, 10.1%)
	Hispanic	**11.3% (6.1%, 16.9%)**	**11.8% (4.4%, 19.8%)**
	Asian	3.3% (-4.1%, 11.1%)	15.5% (-1.8%, 35.8%)
	Other	**27.2% (7.9%, 50.0%)**	**25.0% (2.6%, 52.2%)**
Private	Overall	**10.9% (8.0%, 14.0%)**	**12.0% (8.4%, 15.8%)**
	White	**11.9% (8.4%, 15.5%)**	**12.9% (8.8%, 17.2%)**
	Black	6.6% (-4.9%, 19.6%)	7.0% (-4.9%, 20.4%)
	Hispanic	7.4% (-0.2%, 15.7%)	10.0% (-0.2%, 21.1%)
	Asian	8.5% (-1.9%, 20.0%)	12.4% (-7.7%, 36.8%)
	Other	25.1% (-2.1%, 59.8%)	13.7% (-14.0%, 50.4%)
Medicaid	Overall	**8.3% (3.3%, 13.5%)**	-1.6% (-9.0%, 6.4%)
	White	4.4% (-2.5%, 11.9%)	**-10.0% (-18.8%, -0.3%)**
	Black	14.3% (-2.5%, 34.1%)	1.6% (-16.8%, 24.0%)
	Hispanic	**13.2% (4.0%, 23.2%)**	14.7% (-3.1%, 35.8%)
	Asian	-1.0% (-14.3%, 14.4%)	36.00 (-22.5%, 138.8%)
	Other	**37.0% (2.5%, 83.1%)**	21.3% (-21.9%, 88.4%)

© Nephew LD et al. *JAMA Network Open*.

eTable 7. Estimated incidence rate ratios comparing post-Medicaid expansion waitlisting rates to what would have been observed if the pre-Medicaid expansion era trends had continued in expand and non-expand states

		**Expand States**				**Non-Expand States**			
Insurance	Race	2014	2015	2016	2017	2014	2015	2016	2017
Overall	Overall	1.00<br>(0.96, 1.04)	1.00<br>(0.96, 1.05)	1.01<br>(0.95, 1.07)	1.02<br>(0.95, 1.09)	**0.90<br>(0.85, 0.94)**	0.99<br>(0.93, 1.04)	**1.08<br>(1.01, 1.16)**	**1.19<br>(1.09, 1.30)**
	White	0.99<br>(0.95, 1.04)	0.99<br>(0.93, 1.04)	0.98<br>(0.92, 1.05)	0.98<br>(0.90, 1.06)	**0.83<br>(0.79, 0.88)**	0.94<br>(0.88, 1.00)	1.05<br>(0.97, 1.15)	**1.19<br>(1.07, 1.31)**
	Black	1.08<br>(0.95, 1.23)	1.05<br>(0.90, 1.23)	1.02<br>(0.84, 1.24)	0.99<br>(0.78, 1.26)	0.94<br>(0.81, 1.09)	0.93<br>(0.77, 1.11)	0.91<br>(0.73, 1.14)	0.90<br>(0.68, 1.18)
	Hispanic	0.98<br>(0.90, 1.08)	1.04<br>(0.94, 1.16)	1.11<br>(0.97, 1.26)	1.17<br>(1.00, 1.38)	1.02<br>(0.89, 1.17)	1.10<br>(0.94, 1.29)	1.19<br>(0.98, 1.45)	**1.28<br>(1.01, 1.64)**
	Asian	0.96<br>(0.82, 1.11)	0.97<br>(0.81, 1.15)	0.98<br>(0.79, 1.22)	0.99<br>(0.76, 1.29)	**1.49<br>(1.05, 2.12)**	**1.67<br>(1.10, 2.53)**	**1.87<br>(1.12, 3.12)**	**2.09<br>(1.11, 3.92)**
	Other	1.06<br>(0.77, 1.46)	1.15<br>(0.79, 1.68)	1.25<br>(0.79, 1.99)	1.36<br>(0.77, 2.39)	1.48<br>(0.97, 2.24)	**1.89<br>(1.16, 3.08)**	**2.42<br>(1.33, 4.39)**	**3.10<br>(1.50, 6.42)**
Private	Overall	1.02<br>(0.97, 1.07)	1.05<br>(0.99, 1.11)	**1.08<br>(1.00, 1.16)**	**1.11<br>(1.01, 1.22)**	1.04<br>(0.98, 1.11)	**1.12<br>(1.04, 1.21)**	**1.20<br>(1.10, 1.32)**	**1.29<br>(1.15, 1.45)**
	White	1.03<br>(0.97, 1.09)	1.06<br>(0.99, 1.14)	**1.10<br>(1.00, 1.20)**	**1.13<br>(1.02, 1.26)**	1.04<br>(0.96, 1.12)	**1.12<br>(1.02, 1.22)**	**1.20<br>(1.08, 1.34)**	**1.30<br>(1.14, 1.48)**
	Black	1.17<br>(0.96, 1.41)	1.10<br>(0.88, 1.38)	1.04<br>(0.79, 1.38)	0.99<br>(0.70, 1.39)	0.90<br>(0.72, 1.12)	0.91<br>(0.70, 1.18)	0.92<br>(0.67, 1.27)	0.94<br>(0.63, 1.39)
	Hispanic	0.93<br>(0.81, 1.06)	0.96<br>(0.82, 1.12)	0.99<br>(0.81, 1.20)	1.02<br>(0.80, 1.30)	1.06<br>(0.87, 1.29)	1.14<br>(0.91, 1.44)	1.24<br>(0.93, 1.65)	1.34<br>(0.94, 1.91)
	Asian	0.98<br>(0.79, 1.21)	1.05<br>(0.82, 1.34)	1.12<br>(0.83, 1.52)	1.20<br>(0.83, 1.74)	**1.88<br>(1.21, 2.91)**	**2.04<br>(1.20, 3.48)**	**2.22<br>(1.14, 4.31)**	**2.42<br>(1.08, 5.43)**
	Other	1.10<br>(0.68, 1.79)	1.20<br>(0.68, 2.13)	1.31<br>(0.65, 2.65)	1.43<br>(0.60, 3.39)	1.22<br>(0.67, 2.21)	1.47<br>(0.73, 2.94)	1.77<br>(0.75, 4.15)	2.12<br>(0.75, 6.04)
	Overall	**1.15<br>(1.05, 1.25)**	**1.20<br>(1.08, 1.33)**	**1.26<br>(1.10, 1.43)**	**1.31<br>(1.12, 1.54)**	0.95<br>(0.82, 1.09)	0.92<br>(0.78, 1.09)	0.90<br>(0.73, 1.11)	0.88<br>(0.68, 1.13)

© Nephew LD et al. *JAMA Network Open*.

		Expand States				Non-Expand States			
Insurance	Race	2014	2015	2016	2017	2014	2015	2016	2017
Medicaid	White	1.10 (0.97, 1.25)	1.12 (0.96, 1.30)	1.13 (0.94, 1.36)	1.15 (0.91, 1.44)	0.88 (0.73, 1.05)	**0.79 (0.64, 0.97)**	**0.70 (0.55, 0.91)**	**0.63 (0.46, 0.87)**
	Black	1.04 (0.79, 1.36)	1.09 (0.79, 1.50)	1.13 (0.76, 1.69)	1.19 (0.73, 1.94)	0.75 (0.52, 1.09)	0.71 (0.46, 1.10)	0.68 (0.40, 1.16)	0.64 (0.33, 1.24)
	Hispanic	**1.26 (1.07, 1.48)**	**1.39 (1.15, 1.69)**	**1.54 (1.21, 1.95)**	**1.70 (1.27, 2.28)**	1.27 (0.90, 1.78)	**1.54 (1.04, 2.29)**	**1.86 (1.15, 3.03)**	**2.26 (1.25, 4.09)**
	Asian	1.17 (0.87, 1.57)	1.15 (0.81, 1.63)	1.13 (0.73, 1.74)	1.10 (0.65, 1.88)	1.58 (0.46, 5.41)	2.27 (0.55, 9.26)	3.25 (0.59, 17.95)	4.65 (0.58, 37.40)
	Other	1.05 (0.58, 1.91)	1.33 (0.67, 2.64)	1.68 (0.72, 3.90)	2.12 (0.76, 5.94)	2.19 (0.85, 5.65)	2.69 (0.88, 8.26)	3.31 (0.83, 13.14)	4.07 (0.76, 21.86)

© Nephew LD et al. *JAMA Network Open*.

eTable 8. Estimated incidence rate ratios comparing post-Medicaid expansion waitlisting rates to what would have been observed if the pre-Medicaid expansion era trends had continued in expand and non-expand states in patients without hepatitis-C virus

		**Expand States**				**Non-Expand States**			
Insurance	Race	2014	2015	2016	2017	2014	2015	2016	2017
Overall	Overall	1.04 (0.99, 1.09)	**1.12 (1.05, 1.18)**	**1.20 (1.12, 1.29)**	**1.29 (1.18, 1.41)**	**0.91 (0.86, 0.97)**	1.04 (0.97, 1.12)	**1.19 (1.09, 1.30)**	**1.36 (1.22, 1.51)**
	White	1.05 (0.99, 1.11)	**1.11 (1.04, 1.20)**	**1.18 (1.08, 1.29)**	**1.26 (1.13, 1.40)**	**0.86 (0.80, 0.92)**	1.00 (0.92, 1.09)	**1.17 (1.05, 1.29)**	**1.36 (1.20, 1.55)**
	Black	1.13 (0.93, 1.36)	1.20 (0.96, 1.51)	1.29 (0.97, 1.70)	1.38 (0.98, 1.94)	0.91 (0.74, 1.11)	0.92 (0.73, 1.16)	0.93 (0.69, 1.25)	0.94 (0.66, 1.35)
	Hispanic	1.02 (0.90, 1.14)	1.13 (0.98, 1.30)	**1.26 (1.06, 1.49)**	**1.40 (1.14, 1.73)**	1.07 (0.90, 1.27)	1.20 (0.98, 1.46)	**1.34 (1.04, 1.71)**	**1.50 (1.11, 2.02)**
	Asian	0.93 (0.78, 1.11)	0.96 (0.78, 1.18)	0.99 (0.77, 1.28)	1.02 (0.75, 1.40)	1.46 (0.98, 2.19)	**1.69 (1.04, 2.74)**	**1.95 (1.07, 3.54)**	**2.25 (1.09, 4.66)**
	Other	1.17 (0.78, 1.76)	1.49 (0.92, 2.41)	**1.90 (1.05, 3.43)**	**2.41 (1.17, 4.99)**	1.14 (0.70, 1.87)	1.43 (0.80, 2.54)	1.79 (0.88, 3.62)	2.23 (0.94, 5.28)
Private	Overall	**1.11 (1.04, 1.18)**	**1.23 (1.14, 1.33)**	**1.37 (1.24, 1.50)**	**1.51 (1.35, 1.70)**	**1.09 (1.01, 1.18)**	**1.23 (1.12, 1.35)**	**1.37 (1.22, 1.54)**	**1.54 (1.33, 1.77)**
	White	**1.14 (1.06, 1.23)**	**1.28 (1.17, 1.40)**	**1.43 (1.28, 1.60)**	**1.60 (1.40, 1.84)**	1.09 (1.00, 1.20)	**1.23 (1.11, 1.37)**	**1.39 (1.22, 1.59)**	**1.57 (1.34, 1.85)**
	Black	1.31 (0.99, 1.72)	**1.39 (1.01, 1.92)**	1.49 (1.00, 2.21)	1.58 (0.97, 2.58)	0.92 (0.69, 1.22)	0.98 (0.71, 1.37)	1.05 (0.70, 1.58)	1.12 (0.68, 1.86)
	Hispanic	0.98 (0.82, 1.17)	1.06 (0.86, 1.30)	1.13 (0.88, 1.47)	1.22 (0.89, 1.67)	1.12 (0.89, 1.42)	1.24 (0.93, 1.64)	1.36 (0.96, 1.93)	1.49 (0.97, 2.30)
	Asian	0.91 (0.71, 1.15)	0.98 (0.75, 1.30)	1.07 (0.76, 1.50)	1.16 (0.76, 1.76)	**1.91 (1.16, 3.14)**	**2.14 (1.17, 3.93)**	**2.41 (1.14, 5.10)**	**2.70 (1.08, 6.76)**
	Other	1.22 (0.66, 2.26)	1.53 (0.74, 3.15)	1.91 (0.79, 4.65)	2.39 (0.81, 7.07)	0.84 (0.43, 1.66)	0.96 (0.43, 2.12)	1.09 (0.41, 2.91)	1.24 (0.37, 4.13)
Medicaid	Overall	1.11 (0.99, 1.24)	**1.20 (1.05, 1.38)**	**1.30 (1.10, 1.54)**	**1.41 (1.14, 1.73)**	0.89 (0.74, 1.07)	0.88 (0.71, 1.08)	0.86 (0.66, 1.12)	0.85 (0.61, 1.17)
	White	1.06 (0.90, 1.25)	1.11 (0.91, 1.35)	1.16 (0.90, 1.49)	1.21 (0.89, 1.64)	0.82 (0.65, 1.03)	**0.73 (0.56, 0.96)**	**0.66 (0.47, 0.92)**	**0.59 (0.39, 0.90)**
	Black	1.01 (0.68, 1.48)	1.15 (0.73, 1.82)	1.31 (0.75, 2.32)	1.50 (0.75, 3.01)	0.78 (0.49, 1.25)	0.79 (0.46, 1.37)	0.81 (0.41, 1.59)	0.82 (0.36, 1.89)

© Nephew LD et al. *JAMA Network Open*.

		Expand States				Non-Expand States			
Insurance	Race	2014	2015	2016	2017	2014	2015	2016	2017
	Hispanic	1.18 (0.96, 1.45)	**1.33 (1.04, 1.71)**	**1.51 (1.11, 2.06)**	**1.71 (1.17, 2.50)**	1.18 (0.78, 1.79)	1.36 (0.84, 2.21)	1.56 (0.86, 2.84)	1.79 (0.86, 3.72)
	Asian	1.25 (0.89, 1.77)	1.24 (0.82, 1.88)	1.23 (0.74, 2.06)	1.22 (0.65, 2.29)	1.57 (0.38, 6.45)	2.13 (0.41, 10.94)	2.90 (0.39, 21.30)	3.94 (0.35, 44.87)
	Other	0.79 (0.38, 1.62)	1.08 (0.48, 2.45)	1.48 (0.55, 4.01)	2.03 (0.60, 6.84)	1.30 (0.44, 3.87)	1.58 (0.44, 5.66)	1.91 (0.40, 9.19)	2.32 (0.34, 15.81)

© Nephew LD et al. *JAMA Network Open*.
